# Supplementary figures and images for: Distribution of psychrophilic microorganisms in a beef slaughterhouse in Japan after cleaning
Source: PLoS One. 2022 Aug 3;17(8):e0268411. doi: 10.1371/journal.pone.0268411 (PMC9348744; doi:10.1371/journal.pone.0268411)

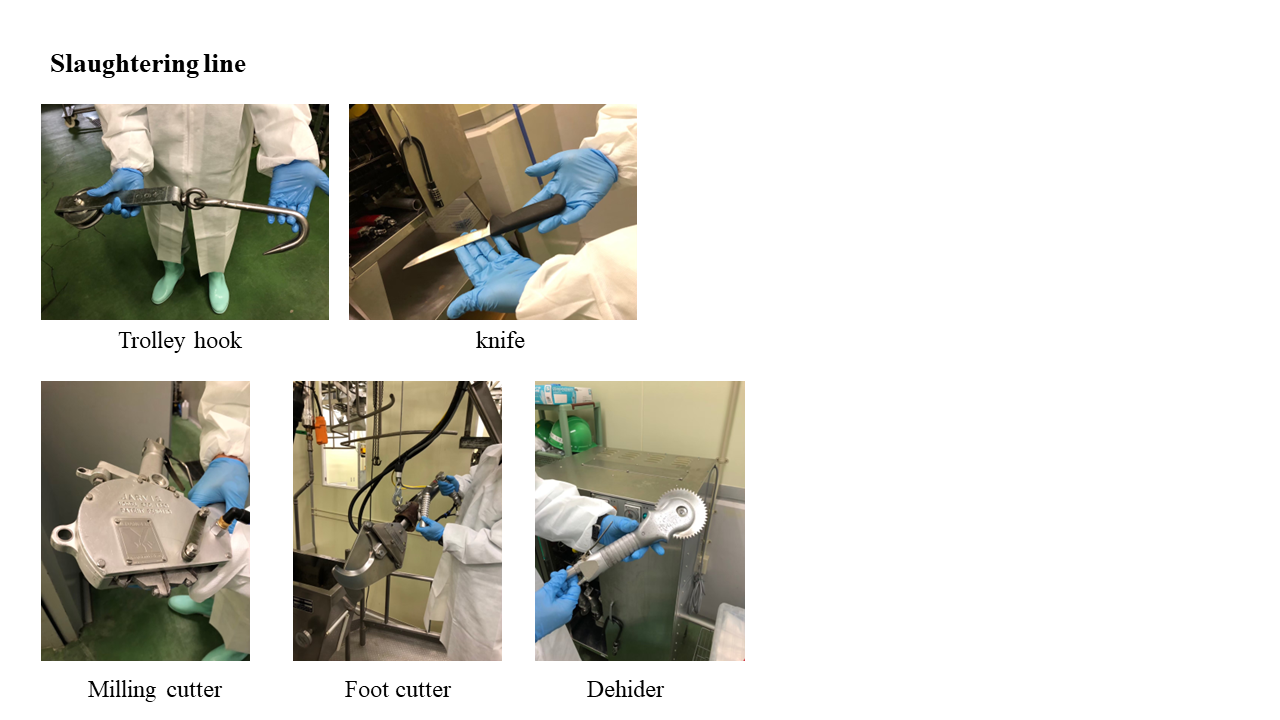

Supplement: S1 Fig — (TIF) [file pone.0268411.s001.tif]

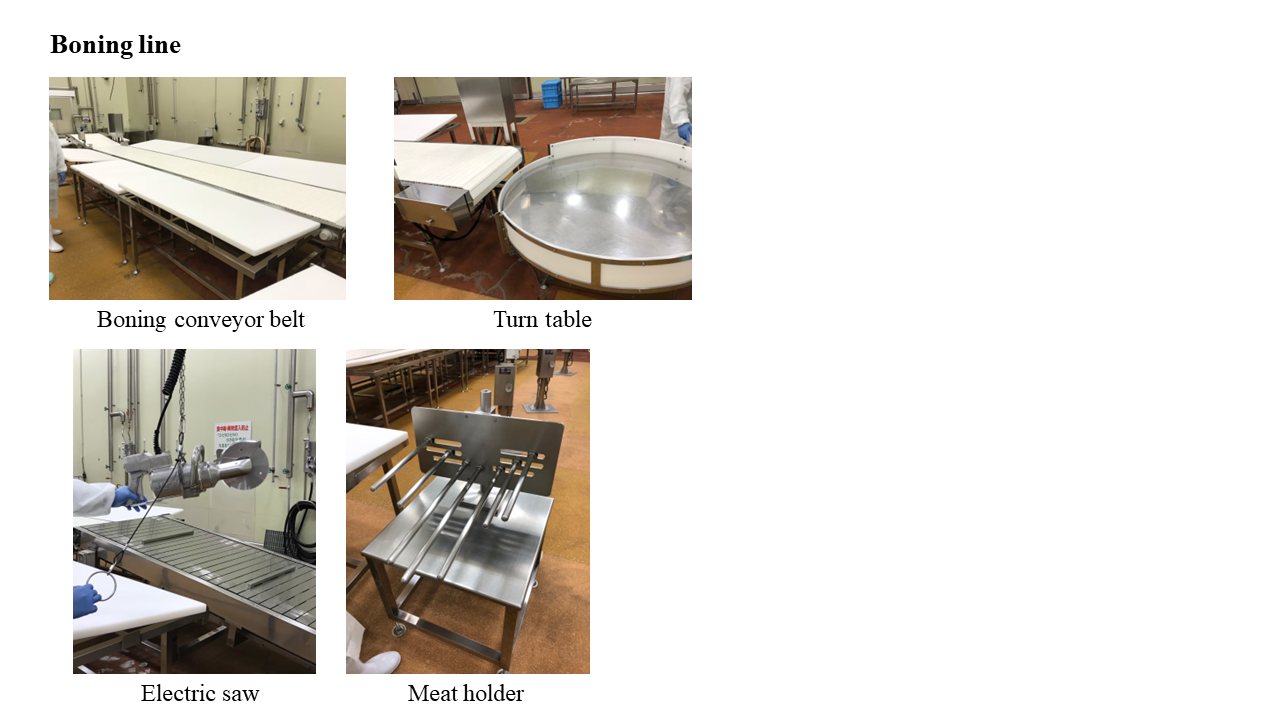

Supplement: S2 Fig — (TIF) [file pone.0268411.s002.tif]
