# Supplementary material for: Distribution of psychrophilic microorganisms in a beef slaughterhouse in Japan after cleaning
Source: PLoS One. 2022 Aug 3;17(8):e0268411. doi: 10.1371/journal.pone.0268411 (PMC9348744; doi:10.1371/journal.pone.0268411)
Supplement: Attachment — Submitted filename: Response to Reviewers.docx [file pone.0268411.s003.docx]

**Response to Reviewers**

Thank you for inviting us to submit a revised draft of our manuscript entitled, “Distribution of psychrophilic microorganisms in the beef slaughterhouse after cleaning” to PLOS ONE. We also appreciate the time and effort you and each of the reviewers have dedicated to providing insightful feedback on ways to strengthen our paper. Thus, it is with great pleasure that we resubmit our article for further consideration. We have incorporated changes that reflect the detailed suggestions you have graciously provided. We also hope that our edits and the responses we provide below satisfactorily address all the issues and concerns the reviewers have noted.

**Dear Reviewer #1:**

- **Abstract**

**L19: give details on the methods used to determine microbial contamination**

(Response)

Thank you for the suggestion.

As recommended, I added detailed information about the microbial investigation.

(Page 2, Line 18–21): The processing lines and equipment used in slaughtering and boning were swabbed, and the microbial count was determined by the culture method using a TSA and MRS medium and Chromocult® Coliform agar incubated at 15ºC and 37ºC, respectively.

**L22: “Petri dish”… how did you select the colonies?**

(Response)

Thank you for your question. I investigated the predominant microbial species attached to the equipment’s surface. I randomly selected four colonies from one plate. I added detailed information to the original manuscript as follows:

(Page 2, Line 23–25) Next, four colonies were randomly isolated from the one petri dish used for the bacterial count measurement to identify the predominant microbial species of the microorganisms attached to each equipment.

**L24: WGS or other sequencing technology? If you also looked for yeasts, you need to correct “the bacterial species” that you mention before.**

(Response)

Thank you for your accurate instruction. I used sanger sequencing technology and I added the information to the manuscript (Page 2, Line 26). Also, I changed “the bacterial species” to “the microbial species” (Page 2, Line 25).

**L27: leave this conclusion for the end… where do you think the overall high contamination comes from and how did you investigate environmental contamination?**

(Response)

Thank you for your question. This study did not investigate environmental contamination. However, the Discussion section cites that these yeasts and bacteria were isolated from soil and livestock (Page 20, Line 286–92).

- **Introduction**

**L41: what about Japan? What is the status quo? You keep mentioning the international point-of-view-, but since your samples were collected in Japan (an in only one facility), you should start from there and then compare with the international practices.**

(Response)

Thank you for your insightful feedback. In fact, in Japan, introducing HACCP became mandatory in slaughterhouses from June 2021. I should have included information about this in the manuscript. Thank you for pointing it out. I modified the document as follows:

(Page 4, Line 42–45) Process control by the Hazard Analysis and Critical Control Point (HACCP) system is one measure to prevent the contamination of carcasses by such harmful bacteria. In Japan, following the EU and the United States, introducing the HACCP system in slaughterhouses became mandatory from June 2021.

**L62: cannot be stopped? Then why do we do it?**

(Response)

I'm sorry that my previous sentence lacked an explanation. Beef chilled shipping exports can be transported while preserving beef quality compared to frozen conditions. However, low-temperature-growing microorganisms can grow and cause quality degradation in beef. It is important to reduce microorganisms that adhere to beef as much as possible to prevent such a scenario. I corrected the sentence as follows:

(Page 5, Page 63–65) However, long transportation periods can allow some low-temperature-growing microorganisms to grow and degrade beef quality.

**L66: remove “to zero”**

(Response)

Thank you for your instruction. I remove “to zero”.

- **Materials and Methods**

**L86: “Materials”**

(Response)

I apologize for simple mistake. I corrected it.

**L89: why not before?**

(Response)

Thank you for your critical question.

This study aimed to investigate the distribution of psychrophilic microorganisms in the equipment used in the slaughtering and boning lines and identify the equipment on which microbial contamination is likely to remain after washing. Therefore, we targeted the equipment after cleaning. By feeding back information about difficult-to-clean equipment to the field workers, they can select an appropriate cleaning method and expect further improvement in hygiene management.

**L93: what is the wiping inspection?**

(Response)

I apologize. My previous sentence was not appropriate. I changed the sentence as follows:

(Page 7, Line 89-90) Cleaning was carried out after each line was used, and the equipment was wiped the day after cleaning.

**L87: Please provide a schematic figure with all sampling points.**

(Response)

Thank you for your suggestion. This study aimed to investigate the distribution of psychrophilic microorganisms that adhere to equipment after cleaning, and we do not discuss the transmission of microorganisms in slaughterhouse. Therefore, I decided that the schematic figure should not be included. Instead, we added a photo of the equipment targeted in this study to the supplementary figure. In the future, when conducting research such as strain typing to explore the dynamics of microorganisms in the slaughterhouse, I would like to attach a schematic figure as you suggested. Thank you for your valuable opinion.

**L108: what is the difference between these media e.g., what are they selective for?**

(Response)

Thank you for your question. I added the information regarding the media as follows:
(Page 8, Line 110–114) The number of bacteria in each swab sample was measured on three types of media: trypticase soy agar (TSA; Becton, Dickinson and Company, Franklin Lakes, NJ, USA) for general bacteria, de Man, Rogosa, and Sharpe (MRS) agar (Merck KGaA, Darmstadt, Germany) for lactic acid bacteria, and Chromocult®︎ Coliform Agar (Merck) for coliform bacteria and *E. coli*.

**L110: which dilution did you plate?
thank you for your question. I added the information as follows:**

(Response)

(Page 8, Line 108-109) The swab sample was diluted ten-fold with saline solution, and 100 µL of each original solution and diluent were spread onto each agar medium.

**L136: “)” missing**
(Response)

Thank you for your polite point. I corrected it.

**L120: please split this in two subheadings**
(Response)

Thank you for your suggestion. I split this paragraph in two subheadings.

- **Results and Discussion**

**L164: how did you calculate the adhesion rate?**

(Response)

Thank you for your question.

We sampled the slaughtering and boning lines six and seven times, respectively. Adhesion rate (positive rate in Tables 1 and 2) indicates the ratio of the number of times that the number of bacteria above the detection limit was confirmed. Due to the previous sentence confusion, I modified it as follows:

(Page 11, Line 161-164) Among the equipment used in the slaughtering line, the brisket saw (handle side) and trolley hook were contaminated with microorganisms in the majority of trials, and the positive rate of microorganisms was 66.7% (four trials were positive among six samplings)**.**

**How do you explain the distribution of *Pseudomonas* sp.?**

(Response)

Since *Pseudomonas* was widely distributed in this slaughterhouse, we presumed that the original contamination from the living body was large. In other studies I am conducting, *Pseudomonas* accounts for more than 80% of the flora on the carcass surface during the slaughter process. Hence, we assumed that *Pseudomonas* from the carcass contaminates the equipment and remains there after cleaning.

**Additionally, do you mean sp. or spp.?**

(Response)

"sp." is used as the singular and "spp." is used as the plural. In this manuscript, all are written as "sp.".

**Dear Reviewer #2:**

The objective of this study was to investigate the abundance and distribution of psychrophilic microorganisms associated with spoilage in beef slaughterhouse environments after cleaning. The topic is interesting, but there are two relevant aspects that weaken the work. The count of psychrophilic was carried out in different sectors of a slaughterhouse, but this does not imply that they are associated with meat contamination and its subsequent alteration. Since the psychrophilic microorganisms present in the meat were not identified, it was not possible to associate whether the contamination found in the slaughterhouse environment had any relevance to the meat quality. On the other hand, the number of colonies isolated for later identification was low and it is not possible to establish the diversity of microorganisms present or their load on the equipment.

(Response)

Thank you for your insightful comments.

In past studies, we conducted a long-term preservation test of beef carcasses slaughtered and dismantled at this slaughterhouse (Dynamics of microbiota in Japanese Black beef stored for a long time under chilled conditions. Food microbiology, 2021, 100: 103849). That study reported that *Pseudomonas* sp. was the predominant bacterial group in the meat when stored in aerobic packaging. It is well reported that the *Pseudomonas* genus is a psychrophilic spoilage-causing bacterium. This study revealed that *Pseudomonas* sp. was widely distributed in the equipment used in this slaughterhouse. From these results, we believe that the psychrophilic microorganisms identified in this study may adhere to the meat and are associated with spoilage. I added the following to the Discussion section:

(Page 25, Line 333–339) Previously, we conducted a long-term preservation test of beef carcasses slaughtered and dismantled at this slaughterhouse (8). Under aerobic storage conditions, the bacterial counts were reached at 8–9 log CFU/g, and *Pseudomonas* sp. predominated the beef microbiota at nine weeks. Numerous studies reported that *Pseudomonas* sp. is related to meat spoilage under low-temperature conditions (20–23). Hence, we consider it necessary to remove *Pseudomonas* sp. from the equipment by washing because of its putrefactive activity.

I selected four colonies for later identification. Certainly, the resolution is low for investigating bacterial diversity, but it is sufficient for investigating the predominant bacterial group. There were many test plots in which this study identified all four strains of the four selected colonies as belonging to the same genus.

**Line 30: It is concluded that, despite the cleaning system used in the slaughterhouse, the concentration of psychrophilic was "very high". However, no parameters are given to support that this load was "very high".**

(Response)

Thank you for your opinion. As you mentioned, I should not use “highly contaminated”. I modified the sentence as follows:

(Page 2, Line 31–32) Despite the cleaning operations, this study identified some equipment contaminated with microorganisms.

**The introduction seems to be very long. Authors should focus on the most important aspects that support their work.**

(Response)

Thank you for your valuable opinion. PLOS ONE has no limit on the number of characters in the manuscript, and few people are familiar with the hygiene management of beef slaughterhouses, so I wrote the introduction with this in mind. I believe that all the paragraphs are important in explaining the background of this study. Some sentences were changed with the reader’s understanding in mind (Page 4, Line 42–45; Page 5, Line 63–65; Page 6, Line 80-82). It would be helpful if you could proceed without cutting the content.

**Lines 81-82: "The ultimate goal of this study was to reduce contamination of carcasses by microorganisms from slaughter processing lines and equipment". This objective was not addressed in this work.**

(Response)

Thank you for your critical opinion. The previous sentence was a leap forward. I modified the sentence as follows:

(Page 6, Line 80-82) The purpose of this study was to understand the actual contamination of low-temperature microorganisms in the equipment used in the slaughtering and boning lines.

**Lines 190-192: "If psychrophilic bacteria can be removed entirely from the equipment and lines through daily cleaning, it is considered that the adhesion of microorganisms to the carcass can be reduced, allowing the beef’s expiration date to be extended". It is quite obvious that if microorganisms are completely removed from equipment and surfaces in slaughterhouses, contamination of meat will be greatly reduced. However, the goal of a cleaning system is not sterilization but the reduction to acceptable levels. Therefore, observed microorganisms concentration should be compared with expected concentrations to determine their impact. This is not addressed in this work.**

(Response)

The cleaning system needs to reduce the number of bacteria on the equipment’s surface to below the detection limit. In fact, at this facility, they perform a wiping inspection of the equipment after cleaning once a week, targeting mesophilic bacteria (35–37ºC). If the number of bacteria is measured, they sanitize the equipment again. This study investigated psychrophilic bacteria and the number of *Escherichia coli* and coliform bacteria, which are mesophilic bacteria. We measured the number of psychrophilic bacteria but not mesophilic bacteria. From this result, it was assumed that psychrophilic bacteria are more likely to remain on the instrument’s surface than mesophilic bacteria. However, no instrument had a 100% detection rate of psychrophilic bacteria on the instrument’s surface after cleaning, indicating that the number of psychrophilic bacteria could be reduced to below the detection limit in all equipment. Therefore, this study regarded the value as unacceptable when the number of bacteria in the equipment after cleaning was above the detection limit.

**Lines 275-276: It is M&M.**

(Response)

Thank you for your instruction. I deleted the sentence.

**Line 321: It just remains to check if the microorganisms present in the meat are the same as those identified in the equipment and surfaces. So, It seems to be a hypothesis (logical) or a speculation.**

(Response)

Thank you for your opinion. As you instructed, I modified the sentence as follows:

(Page 25, Line 316-319) This study did not investigate whether microorganisms present in the meat are the same as those identified on the equipment and surfaces. However, since the edge side of the saw comes into direct contact with the carcass, there is a high possibility that microbial contamination will spread from the carcass.
