# Supplementary material for: Distribution of psychrophilic microorganisms in a beef slaughterhouse in Japan after cleaning
Source: PLoS One. 2022 Aug 3;17(8):e0268411. doi: 10.1371/journal.pone.0268411 (PMC9348744; doi:10.1371/journal.pone.0268411)
Supplement: Attachment — Submitted filename: renamed_3070f.docx [file pone.0268411.s004.docx]

**Response to Reviewers**

Thank you for inviting us to submit a revised draft of our manuscript entitled, “Distribution of psychrophilic microorganisms in a beef slaughterhouse in Japan after cleaning” to PLOS ONE. We also appreciate the time and effort you and each of the reviewers have dedicated to providing insightful feedback on ways to strengthen our paper. Thus, it is with great pleasure that we resubmit our article for further consideration. We have incorporated changes that reflect the detailed suggestions you have graciously provided. We also hope that our edits and the responses we provide below satisfactorily address all the issues and concerns the reviewers have noted.

**Dear Reviewer #1:**

Thank you for your detailed suggestions regarding the Abstract. Thanks to these, the text has been vastly improved. With regard to the Discussion section, because there are currently no published reports of studies similar to the one we have carried out, it was difficult to compare and discuss our results with other findings side by side. However, we have deepened the discussion as much as possible. Thank you for pointing this out.

**Title: replace “the” by “a” … in Japan after cleaning**

(Response)

Thank you for the suggestion. As recommended, I modified the title.
**Abstract:**
**L19: remove “by the culture method”**

(Response)

Thank you for your instruction. I removed “by the culture method”
**L21: remove “in most trials”, otherwise you need to give before how many trials were conducted**

(Response)

Thank you for your instruction. I removed “in most trials”

**L22: remove “confirmed to be”**

(Response)

Thank you for your instruction. I removed “confirmed to be”
**L24: remove “one”**

(Response)

Thank you for your instruction. I removed “one”
**L26: Sanger, not sanger**
(Response)

Thank you for your correction. I corrected it.

**Can you give the % of contamination or the number of samples e.g., (10/24) where you found the microorganisms? Gives an idea of the prevalence of contamination, which is an easy and interesting info to extract directly from the Abstract**
(Response)

Thank you for your suggestion. I have added the information about the percentages of contamination, as indicated below. Thanks to you, the Abstract text is now better.

**Page 2, Lines 21–25:** As a result, the brisket saw (handle side) and trolley hook were the most heavily contaminated with microorganisms, with each having a microbial adhesion rate of 66.7%. The microbial adhesion rates of the apron and milling cutter (edge side) were 50%, respectively, and those of the foot cutter (edge and handle side), splitting saw (edge side), and knife (handle side) were 33.3%, respectively.

**Results and discussion:
This section is very poorly discussed. There is indeed a lot of detail given, but barely any discussion with the available literature. This is not a report, it is a research article.**
(Response)

Thank you for your helpful suggestions. To the best of our knowledge, there are no published papers on studies that have investigated the distribution of psychrophilic microorganisms on different slaughterhouse equipment after cleaning. Therefore, a discussion based on available literature findings was difficult. However, we have deepened our discussion on the following aspects: why contamination was high in the specific equipment (Lines: 206–218, 238–246, 268-271), the type of bacteria attached (Lines: 286–295, 353–361), and which of those bacteria are involved with putrefactive risks (Lines: 330-342, 361-364). Additionally, in response to your suggestions, we have added following discussion section regarding yeast contamination of food industry and yeast susceptibility against disinfectant. Thank you for your valuable opinion.

**Page 27, Line 364-373:** In the past, the distribution of spoilage yeasts was investigated with instruments in wineries, bakery industries, breweries, yogurt factories or goat cheese industries. The processed meats industry also investigated the distribution of yeast across the instruments used in the industry and identified the surface of facilities, including room equipment and production materials, as the main source of yeast contamination of cured meat [35]. In particular, *Candida zeylanoides* is frequently isolated from cured meat, and considered predominant in the raw material, fresh meat. In this study, *Candida zeylanoides* was frequently isolated from the equipment used in the boning line, suggesting that the slaughterhouse, which is the most upstream food distribution center, may be a source of yeast contamination of meat.

**Page 28, Line 375-385:** Microbial contamination was concentrated on the handle side of the electric saw, where microbes were frequently detected in multiple trials. The concentration of disinfectants, required to remove yeast, was higher than that of food-related bacteria [36]. Salo et al. (2005) demonstrated that alcohol-based disinfectants were most effective in decontaminating yeast isolates [37]. In addition, surfactant-based and peroxide-based disinfectants were effective against floating yeast cells, while biofilm carrier tests reported the effectiveness of chlorine-based foam cleaners. On the other hand, disinfectants containing chlorine and persulfates have failed to kill yeast cells in both suspensions and biofilm formation. As the susceptibility of bacteria and yeast to disinfectants is different, a thorough review of cleaning methods for yeast contaminated equipment is warranted.

**L335-339: Pseudomonas should be italicized. This section also reflects my previous comment: it should be spp., as you would expect several species of Pseudomonas to be found, not just one specific species (for those cases, use sp., when you are referring to a specific Pseudomonas).**

(Response)

Thank you for pointing out these errors. I apologize for overlooking the italicization of bacterial names and this has since been corrected. Also, as you pointed out, when multiple *Pseudomonas* species are being described, they are represented as “spp.”

**Dear Reviewer #2:**

**I understand and agree with the authors that Pseudomonas are the most representative populations of psychrophilic microorganisms in meat. However, from this study, you cannot assume that the microorganisms present in the equipment are the same as those on the carcasses. For this, it should resort to molecular studies.**

(Response)

We agree totally with what you say. It is necessary to use molecular typing methods (MLST, RAPD, ribotyping, etc.) to determine whether the bacteria attached to the equipment actually propagate to the carcass surface. With regard to this point, the following has been added in the relevant part as the limitation of this research. Thank you for your valuable feedback. We would like to incorporate your opinions into our Future work.

**Page 26, Lines 344-349:** This study did not investigate whether microorganisms present in the meat are the same as those identified on the equipment and surfaces. To clarify this, strain identification using molecular typing methods, such as RAPD, MLST, and ribotyping, need to be performed [24-29]. Further detailed research should be carried out in the future to determine what kinds of instruments and bacterial species are likely to contaminate the surface of carcasses.
